# Changes over time in prescription practices of pain medications in Switzerland between 2006 and 2013: an analysis of insurance claims

**DOI:** 10.1186/s12913-017-2086-6

**Published:** 2017-02-27

**Authors:** Maria M. Wertli, Oliver Reich, Andri Signorell, Jakob M. Burgstaller, Johann Steurer, Ulrike Held

**Affiliations:** 1 Horten-Centre for patient oriented research and knowledge transfer, University of Zurich, CH-8032 Zurich, Switzerland; 2 Department of Health Sciences, Helsana, Zürichstrasse 130, CH-8600 Dübendorf, Switzerland; 3 Department of General Internal Medicine, Bern University Hospital, University of Bern, CH-3010 Bern, Switzerland

**Keywords:** Pain medications, Opioids, Non-opioids, NSAID, Paracetamol, Metamizole, Insurance claims data

## Abstract

**Background:**

In Europe, scant information is available about prescription practices for pain medications. The aim of this research was to assess changes in prescription rates of non-opioid, weak opioid, and strong opioid medications between 2006 and 2013 in the Swiss population.

**Methods:**

Using insurance claims data covering one-sixth of the Swiss population, we analyzed the numbers of reimbursed pain medications, the number of reimbursements per persons, and the cumulative dose in milligrams. For opioids, the morphine equivalent dose and treatment days were calculated. Data were extrapolated to the dose per day per 100’000 population stratified by age, gender, and canton.

**Results:**

In total, 4’746’942 paracetamol, 2’156’620 NSAIDs or Coxibs, 931’129 metamizole, 1’322’272 weak opioid, and 807’835 strong opioid claims were analyzed. Between 2006 and 2013, the increase in claims per 100’000 persons was 32% for paracetamol, 242% for metamizole, 107% for NSAIDS, 86% for Coxibs, 13% for weak opioids, and 121% for strong opioids. For strong opioids the total MED in mg /100’000 increased by 117%, the treatment days /100’000 by 101%. For strong opioids, fentanyl was most frequently used (increase between 2006 and 2013 by 91% for MED/100’000 persons and 94% treatment days / 100’000) followed by buprenorphine and oxycodone. The highest proportional increase in MED / 100’000 was observed for methadone (+1414%) and oxycodone (+313%). Marked geographical variation was detected in the use of metamizole, paracetamole, and strong opioids in different cantons.

**Conclusion:**

The analysis of insurance claims data provides evidence that the prescription rates for pain medications increased in Switzerland within the last ten years, in particular for metamizole and strong opioids. Furthermore, the prescription rates for metamizole, paracetamol, and strong opioids varied substantially between different cantons in Switzerland.

**Electronic supplementary material:**

The online version of this article (doi:10.1186/s12913-017-2086-6) contains supplementary material, which is available to authorized users.

## Background

Between 1990 and 2010, the number of patients with non-infectious illnesses increased worldwide by more than 40% [1]. As a consequence, the number of patients with chronic pain conditions increased, and the main causes are musculoskeletal disorders, the sequela of injuries, and malignancies [1, 2]. In the 1990s, the World Health Organization (WHO) recommended a stepwise increase of treatment intensity in cancer pain which improved pain control (the WHO pain relief ladder [3, 4]). For mild pain, non-opioids were recommended; for mild to moderate pain, weak opioids (e.g. tramadol, codeine); and for strong pain, strong opioids (e.g. morphine, fentanyl) [4]. Although the stepwise approach is widely accepted, the choice of a specific pain medication is influenced by several factors including comorbidities. Further, the withdrawal of most cox-2 inhibitors from the market reduced the non-opioid based treatment options [5].

Opioids are well established for the relief of acute strong pain, chronic pain in patients with active cancer, or for symptom relief at the end of life. In chronic non-cancer pain, opioids are considered second line drugs because they are usually not more effective than non-opioid pain medications [6, 7] but potentially decrease quality of life and pain control [8, 9]. Despite these limitations, opioids are increasingly prescribed for chronic non-cancer pain and the prescription rates have reached enormous dimensions in some countries [10–12]. According to consumer data, the sale of opioids increased in the US from 96 mg of morphine equivalents per person in 1997 to 710 mg per person in 2010 [6]. The total amount is equivalent to 7.1 kg of opioid medication per 10,000 people, or 5 mg of hydrocodone every 6 h for 45 days for every adult American [6]. In parallel to the increased use of opioids, the risk for unintentional opioid overdose of strong opioids increased [13–16] and resulted in higher hospital admission rates [17]. In Europe, scant information is available about prescription practice for pain medications. However, a study on changes in pain medication use in Scotland showed an increased use of pain medications and an 18-fold increase in the use of strong opioids indicating that also in Europe strong opioids are increasingly used [18].

The aim of this analysis was to describe changes in prescription rates of non-opioid, weak opioid, and strong opioid medications between 2006 and 2013 in age groups and geographical areas in the Swiss population. We hypothesized that the prescription of strong opioids increased exponentially compared to other pain medications.

## Methods

### Study design

Analysis was done of insurance claims data from one of the major health insurers in Switzerland, the Helsana health insurance group. The insurer covers 1.2 million individuals (approximately one-sixth of the Swiss population) in all 26 cantons, and maintains records of all health care invoices including information about prescribed medications. The patient-level linked database provides information on socio-demographic data, health insurance status, prescribed drugs, health care utilization and its associated costs (inpatient, outpatient) as well as the date of death.

### Study population

All administrative claims data of adults (age 18 years and older) who received reimbursement for at least one pain prescription between January 2006 and December 2013 were included in this analysis. All reimbursed medication claims are labeled with a unique code for the pharmaceutical class (see definitions). The code is based on the WHO pharmacological Anatomical Therapeutic Chemical (ATC) classification system [19]. Patients were identified by the ATC codes representing the following pain medications that were included in this analysis: non-opioids, weak opioids, and strong opioids.

### Swiss healthcare system and regulation for opioid prescription

In Switzerland compulsory basic health insurance is universal and covers the population of 8.2 million persons. There are virtually no uninsured residents. The basic health insurance covers a comprehensive benefits package defined by federal authorities and can be supplemented by private/semiprivate insurances that offer additional services [20]. The coverage provided through compulsory, individually purchased health insurance is a comprehensive benefit package that leads to out-of-pocket spending from copayments and deductibles [20]. The amount of copayment depends on the chosen deductible class. Several health care providers and payers are on the market and no centralized registration of prescriptions is available. Further, the 26 Swiss cantons are responsible for the planning and delivery of the health services and thus, the health care system is highly decentralized. Therefore, no centralized opioid use register is available. Opioids cannot be purchased over the counter and for strong opioids a special prescription (a so called “prescription for narcotic substances”) is issued on prescriptions with 3 copies including a unique identification number. One copy remains with the prescribing physician, one with the pharmacy and with the insurance company. While this regulation reduces the risk of abuse, there is no central database that would identify patients with multiple prescriber or other misuse.

### Definitions

Non-opioid pain medications: paracetamol (N02BE01, N02BE51, N02BE71), nonsteroidal anti-inflammatory drugs (NSAIDs, M01AA, M01AB, M01AC, M01AE, M01AG), cox-inhibitors (Coxibs, M01AH), metamizole (N02BB02, N02BB52, N02BB72).

Weak opioids included oral or rectal opioid formulations with a morphine conversion factor of 0.3 or less. ATC codes and substances for weak opioids were: N02AA59 (codeine and combinations), N02AX01 (tilidine), N02AX02 (tramadol), N02AX06 (tapentadol).

Strong opioids: all opioids not defined as weak. The full list of ATC codes, opioid substances, conversion factors, route of administration, and defined daily dose are provided in Additional file 1. In Switzerland hydrocodone (ATC code R05DA03) is not listed by the authorities and not covered by the basic insurance coverage and therefore, not included in this analysis. However, hydrocodone is in rare cases prescribed by physicians and a prescription for narcotic substances is required with the same regulation as described above.

Reimbursed pain medications: Each reimbursement of a pain medication (referred to hereafter as a “claim”) was converted to a total amount of substance by calculating the number of pills per reimbursement × strength of the substance. For each pharmaceutical class of pain medications, the total numbers of claims reimbursed, the average number of claims per person receiving pain medications, and the cumulative dose of milligrams (mg) of the active pharmaceutical substance were calculated and reported for each year between 2006 and 2013. Wherever available we calculate the cumulative dose per drug class: paracetamol, metamizole, weak opioids, strong opioids. For NSAIDs and Coxibs no dose conversion for individual formulations is available. Therefore, we report the cumulative dose per substance (e.g. diclofenac, ibuprofen) and depict selected substances.

Morphine equivalent dose (MED): To account for the different potencies of opioids, the morphine equivalent dose (MED) was calculated for each opioid (weak and strong) as follows: Strength of opioid drug in mg per unit x quantity of units per reimbursed package x number of packages x conversion factor for morphine equivalents. The equianalgesic dose conversions are only estimates and cannot account for individual variability in genetics and pharmacokinetics. Wherever available we used conversion factors provided by the Swiss Agency for Therapeutic Products (Swissmedic, agency comparable to the US Food and Drug Administration, FDA) or the morphine equivalent conversion factor per mg of opioid was based on the CONSORT classification (CONsortium to Study Opioid Risks and Trends [21]). Further, we consulted the literature relevant to the topic and a clinical pharmacologist (See Additional file 1: opioids, examples of brand names, the morphine equivalent conversion factors, and the route of administration). The MED calculation for fentanyl patches is based on the assumption that one patch delivers the dispensed (and bioavailable) mcg per hour over 72 h. The calculation of the total bioavailable MED dose in mg equals (mcg/hour (according to the package reimbursed) x 72 h’ x number of patches per package x number of packages reimbursed x 100 [fentanyl conversion factor])/1000. For example, the total MED in mg for one package containing 10 fentanyl patches that each delivers 12mcg per hour is calculated as follows: 12mcg x 72 h × 10 patches × 100 = 864,000mcg/1000 = 864 mg. For transdermal buprenorphine patches the assumption is that one patch delivers the dispensed (and bioavailable) mcg per hour over 96 h. The total MED dose in milligram equals (mcg/h according to the package reimbursed x 96 h’ x number of patches per package x number of packages reimbursed x 95 [buprenorphine conversion factor]) / 1000.

Defined daily dose (DDD): For strong opioids, treatment days were calculated by using the defined daily dose (DDD). The DDD is provided by the WHO ATC and is based on the assumed average maintenance dose per day for a drug used for its main indication in adults [19]. The WHO ATC/ DDD system allows standardization of drug groupings and a stable drug utilization metric to enable comparisons of drug use between countries [19].

Treatment days in MED: For strong opioids we calculated in addition to total MED the treatment days as follows: total MED per substance/DDD.

Morphine equivalent (MED) per treatment day: Total MED in mg/Total treatment days (for strong opioids).

Pain medication per 100’000 population: Reimbursed pain medications were extrapolated to calculate the amount of each pharmaceutical substance prescribed and the treatment days per 100’000 population.

Opioid substitution programs: Opioid substitution programs: We excluded patients using opioids within a drug substitution program. Since 1999, the insurance companies reimburse the opioids for drug substitution. We excluded diamorphine using the corresponding ATC-code (N07BC06 Diaphin®). Other opioids including morphine (N02AA01 Sevre-Long®), buprenorphine (N07BC01 Subutex®, N02AE01 Temgesic®), and methadone (N07BC02) are used within substitution programs and for pain treatment. For opioid dependency substitution a unique outpatient code is used (Tarmed Position 00.0155: non-physician medication distribution for opioid dependency substitution). We excluded all opioid claims from the analysis for all patients where this unique outpatient code was used at least once during the study period (e.g. in a patient the unique code was identified in the database in 2009 then all opioids reimbursed for this person were excluded).

### Statistical methods

Descriptive statistics included median and interquartile range for the continuous parameters, and percentages for the categorical outcomes. The annual number of claims and the cumulative dose of milligrams of the active pharmaceutical substance were extrapolated to calculate the amount of each pharmaceutical substance prescribed and the treatment days per 100’000 population stratified by age (strata of 5 years), gender, and canton using methods proposed for the analysis of complex survey analysis [22]. The age-dependent use of pain medications was studied by calculating the mean annual dose per patient between 2006 and 2013 in the following predefined age groups: 45 years or younger (reference category), 46 to 65, 66 to 75, 76 to 85, and 86 years and older.

Statistical analysis was performed using the computing environment R, a freely available system for statistical computation and graphics environment (https://www.r-project.org/) [23]. The following software packages were used: DescTools, mvtnorm, foreign, Rcpp, RDCOMClient, and tcltk.

## Results

**Fig. 1:**
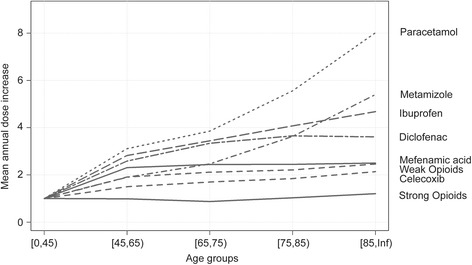
Comparison of the mean annual dose in age groups 45 years and older to patients under the age of 45 years. The mean annual dose increase compared to the reference group of patients age younger than 45 years (1 = 100%). The mean annual dose of each pharmaceutical group was calculated by the mean annual dose per patient in the corresponding age group. The mean annual dose was divided by the mean annual dose in the reference group. ATC codes used: paracetamol (N02BE01, N02BE51, N02BE71), selected NSAIDs (M01AE01 + M01AE51 ibuprofen, M01AB05 + M01AB diclofenac, M01AG01 mefenamic acid), metamizole (N02BB02, N02BB52, N02BB72). Weak opioids (N02AA59, N02AX0, N02AX02, N02AX06), and strong opioids (N02AA01-5, N02AA55, N02AB02, NA02AB03, N02AE01, N02AF02, N07BC1-2)

In 2013, 43% of the total population covered by a Helsana health insurance plan were reimbursed for at least one claim of non-opioid medication, 4.8% for at least one weak opioid claim, and 2.8% for at least one strong opioid claim. In total, 4’746’942 paracetamols, 2’156’620 NSAIDs or Coxibs, 931’129 metamizole, 1’322’272 weak opioids, and 808’751 strong opioid claims were analyzed. Patients receiving NSAIDs and paracetamol were, on average, younger (median age 38 and 39 years) than patients receiving metamizole and opioids (median age 54 and 64 years, Table 1). On average, more pain medication claims were reimbursed for women and their average age was higher than the male subjects. Compared to younger patients (up to age 45) there was an 8-fold increase in the use of paracetamol, and 5.4-fold increase in metamizole use in patients 85 years and older (Fig. 1). The use of NSAIDs increased in all age groups including patients over 85 (diclofenac increased 3.6-fold, ibuprofen 4.5, and mefenamic acid had a 2.5-fold increase). The use in strong opioids did not increase and there was only a slight increase in weak opioids in older age groups compared to the reference group.

**Table 1:** Baseline characteristics for all claims reimbursed between January 2006 and December 2013

	NSAID/Coxibs	Paracetamol	Metamizole	Weak Opioids	Strong Opioids
Number of claims: n total	2'156'620	4'746'942	931'129	1'322'272	807'835
Number of persons: n	693'928	1'098'925	292'403	268'143	121'455
Morphine equivalent: median (IQR)^a^	-	-	-	180 (531)	300 (2680.1)
Male: n (%)	293'011 (42.2)	484'176 (44.1)	120'609 (41.2)	111'888 (41.7)	50'329 (41.4)
Female: n (%)	400'917 (57.8)	614'749 (55.9)	171'794 (58.8)	156'255 (58.3)	71'126 (58.6)
Age: median (IQR)	39 (41)	38 (45)	54 (35)	57 (31)	64 (30)
Age male: median (IQR)	36 (43)	36 (47)	52 (33)	55 (29)	61 (29)
Age female: median (IQR)	41 (40)	40 (43)	55 (37)	59 (32)	67 (31)
German part^b^: n persons (%)	464'237 (66.9%)	766'415 (69.7%)	257'420 (88.0%)	195'149 (72.8%)	101'664 (83.7%)
French part^b^: n persons (%)	195'934 (28.2%)	259'895 (23.6%)	22'835 (7.8%)	57'929 (21.6%)	15'642 (12.9%)
Italian part^b^: n persons (%)	33'757 (4.9%)	72'615 (6.6%)	12'148 (4.2%)	15'065 (5.6%)	4'149 (3.4%)
German part^b^: n claims (%)	1'416'848 (65.7%)	3'059'364 (64.4%)	845'951 (90.9%)	962'477 (72.8%)	671'079 (83.1%)
French part^b^: n claims (%)	637'028 (29.5%)	1'320'874 (27.8%)	54'124 (5.8%)	286'570 (21.7%)	109'364 (13.5%)
Italian part^b^: n claims (%)	102'744 (4.8%)	366'704 (7.7%)	31'054 (3.3%)	73'225 (5.5%)	27'392 (3.4%)

^a^morphine equivalents in milligram

^b^numbers calculated based on the area of residency (canton)

### Change in claims for non-opioid pain medications

Between 2006 and 2013, the use of non-opioid pain medications increased between 25% and 237% and the claims per person rose by 12% (from 2.94 to 3.3, see Table 2 and for more details Additional file 2). The extrapolated claims per 100’000 persons showed an increase for paracetamol, metamizole, NSAIDs and Coxibs by between 32 and 242% (Table 2) with the most substantial increase observed for metamizole (+242%). Compared to NSAIDS Coxibs were less frequently used (in 2013 NSAIDs 28’932 claims/100’000 persons, Coxibs 3’063 claims/100’000 persons).

**Table 2:** Change in claims for non-opioid pain medications, weak opioids, and strong opioids between 2006 and 2013

Non-opioids	2006	2013	% Diff 06-13
Number of persons^a^:	455'845	501'996	10
Age: median (IQR)	47 (41)	48 (41)	
Female: n (%)	265'135 (58.2)	288'289 (57.4)	
Number of claims: n	1'342'454	1'657'420	23
Number of claims / person	2.94	3.3	12
Paracetamol: number of claims	512'762	639'716	25
Metamizole: number of claims	55'000	185'168	237
NSAID^b^: number of claims	174'293	329'075	89
Coxibs: number of claims	22'809	39'743	74
Paracematol: claims / 100'000	40'211	53'062	32
Metamizole: claims / 100'000	4'018	13'729	242
NSAID + Coxibs claims / 100'000	15'634	31'995	105
NSAID claims / 100'000	1'049'968	2'354'922	124
Coxibs claims / 100'000	123'981	249'306	101
Ibuprofen: claims / 100'000	235	395	68
Paracetamol: 1000 mg / 100'000	1'116'931	2'717'129	143
Metamizole: 1000 mg / 100'000	61'074	501'196	721
Weak opioids	2006	2013	% Diff 06-13
Number of persons^a^	53'786	55'604	3
Age: median (IQR)	61 (29)	62 (30)	
Female: n (%)	33'172 (62)	33'629 (60)	
Number of claims#: n	160'517	167'278	4
Number of claims / person	2.98	3.01	1
Number of claims / 100'000	11'957	13'348	13
Total MED in mg	50'339	57'782	15
Total MED in mg/Person	936	1'039	11
Total MED in 1000 mg/ 100'000	3'768	4'655	24
Tramadol in MED 1000 mg/ 100'000	3'046	3'750	23
Codeine MED in 1000 mg/ 100'000	722	587	−19
Tapentadol MED in 1000 mg / 100'000	-	317	-
Strong opioids	2006	2013	% Diff 06-13
Number of persons^a^	16'744	28'509	70
Age: median (IQR)	66 (27)	69 (29)	
Female: n (%)	10'297 (62)	17'570 (62)	
Number of claims: n	64'839	137'458	112
Number of claims / person	3.87	4.82	25
Number of claims / 100'000	4'716	10'419	121
Total MED 1000 mg	146'151	294'320	101
Total MED in mg/Person	8'729	10'324	18
Total treatment days	1'199	2'274	90
Total treatment days/ person	72	80	11
Total MED in 1000 mg/ 100'000	11'011	23'854	117
Total treatment days / 100'000	89'903	180'371	101
Total MED / treatment day	121.9	129.4	6
Morphine: MED 1000 mg / 100'000	2'875	4'648	62
Oxycodone and comb: MED 1000 mg / 100'000	1'237	5'110	313
Fentanyl: MED 1000 mg / 100'000	3'588	6'851	91
Pethidine: MED 1000 mg / 100'000	33	39	18
Hydromorphone: MED 1000 mg / 100'000	548	730	33
Methadone: MED 1000 mg / 100'000	28	424	1414
Buprenorphine: MED 1000 mg / 100'000	2'699	6'049	124
Nalbuphine: MED 1000 mg / 100'000	2	2	0
Morphine: treatment days / 100'000	28'988	46'856	62
Oxycodone: treatment days / 100'000	10'994	45'422	313
Fentanyl: treatment days / 100'000	29'941	57'953	94
Pethidine: treatment days / 100'000	411	491	19
Hydromorphone: treatment days / 100'000	3'657	4'988	36
Methadone: treatment days / 100'000	373	5'645	1413
Buprenorphine: treatment days / 100'000	15'509	18'985	22
Nalbuphine: treatment days / 100'000	31	30	−3
Oral long acting: MED 1000 mg / 100'000	4'409	9'761	121
Oral short acting: MED 1000 mg / 100'000	142	865	509
Parenteral: MED 1000 mg / 100'000	168	328	95
Rectal: MED 1000 mg / 100'000	5	9	80
Sublingual: MED 1000 mg / 100'000	1'243	4'986	301
Transdermal: MED 1000 mg / 100'000	5'045	7'905	57

^a^number of subjects that received reimbursement for one of the corresponding pain medications for a specific year

^b^claims for all NSAID

*MED* morphine equivalent dose

### Change in claims for weak opioid pain medications

Between 2006 and 2013, the use of weak opioids per 100’000 persons in morphine equivalent dose (MED) increased by 24% although the observed rate of claims per person did not increase (Table 2). This increase was mainly due to an increase in tramadol claims (+23%).

### Change in claims for strong opioid pain medications

Between 2006 and 2013, the total number of claims for strong opioids doubled from 64’839 to 137’458 (Table 2). The average claims per person with strong opioids claims increased from 3.87 to 4.82 (+25%). The median age of the population receiving strong opioid prescriptions increased between 2006 and 2013 from 66 to 69 years. Extrapolated to 100’000 persons, the total morphine equivalent dose (MED) increased by 117% and the total treatment days by 101%. Fentanyl was the most frequently prescribed strong opioid in total MED and treatment days per 100’000 (Additional file 3). The proportions of the different strong opioids in 2013 were as follows: fentanyl accounted for 29% of the MED per 100’000 persons, followed by buprenorphine (25%), oxycodone (21%), morphine (19%), hydromorphone (3%), and methadone (3%). Between 2006 and 2013, the treatment days for fentanyl increased by 94% and the total MED by 91% (Table 2, see for more details Additional file 2). The highest proportional increase was observed for methadone (1141% increase in MED/100’000, 1413% increase in treatment days/100’000), which was rarely used in 2006, followed by oxycodone (increased by 313% in MED/100’000 and treatment days/100’000). The use of short-acting formulations increased for oral use by 509%, and for sublingual use by 301%. Most frequently a transdermal route of application of opioids was used.

### Differences in the use of pain medications in different Swiss cantons

**Fig. 2:**
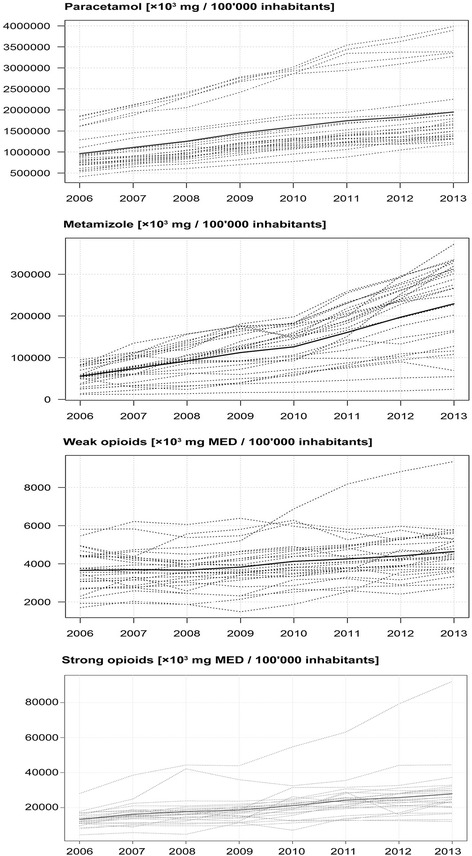
Reimbursement of pain medications in Switzerland between 2006 and 2013. The bold line represents the mean per/100’000 persons, dotted lines represent the areas (cantons) in Switzerland

**Fig. 3:**
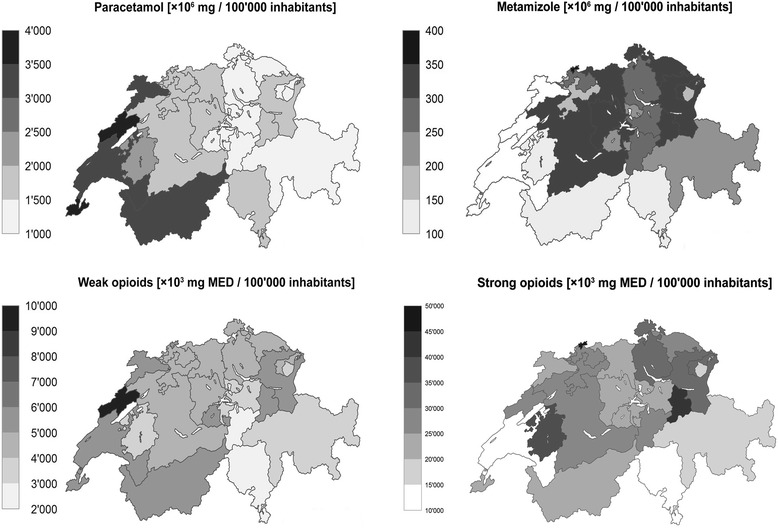
Differences in the use of pain medications in the Swiss cantons in 2013. Graphical representation of the reimbursed claims per 100’000 persons stratified for sex, age, and canton. Copyright geodata Swiss Federal Statistical Office / swisstopo

We found considerable differences in the use of pain medications across the Swiss cantons (Fig. 2). The most pronounced differences between Swiss cantons were identified in the use of metamizole (Fig. 3). In 2013, it was less used in the French and Italian speaking parts of Switzerland and mainly used in the German speaking areas. Paracetamol and weak opioids were more frequently used in the French speaking part. In 2013, strong opioids were most often used in the cantons of Jura, Basel-Stadt, Nidwalden, and Glarus (see Fig. 3, for a comparison of the use of strong opioids in 2006, depicted in Additional file 4). Between 2006 and 2013, the most pronounced increases in the use of strong opioids were detected in the cantons of Jura (+260%), Fribourg (+270%), Basel-Stadt (+219%), Uri (+220%), and Schaffhausen (+201%). as shown in Fig. 3 and Additional file 4. The increases in strong opioid use were lowest in the cantons of Nidwalden (+44%), Valais (+48%), and Zug (+49%).

## Discussion

Between 2006 and 2013, we found an increase in claims per 100’000 persons in almost all pain medications. The increase was most pronounced for metamizole (+324%), NSAIDS (+124%), Coxibs (+101%), and strong opioids (+70%). While fentanyl was the most frequently used strong opioid, we observed the highest proportional increase for methadone (+1414%), followed by oxycodone (+313%). The use of short-acting forms including the oral and sublingual formulation of strong opioids increased markedly. In patients 85 years of age and older, we found an increased prescription of all pain medications including NSAIDs.

We found pronounced geographical differences in the prescriptions for metamizole, paracetamol, and strong opioids. Metamizole was mainly and increasingly prescribed in the German speaking part of Switzerland. Paracetamol and weak opioids were more frequently used in the French speaking part. The prescription for strong opioids increased between 2006 and 2013, most frequently in the cantons of Jura, Fribourg, Basel-Stadt, Uri, and Schaffhausen.

### Results in light of the literature

After the recall of the popular coxib rofecoxib in 2005, an analysis of consumer data in Switzerland found an increase in the use of all non-opioid and opioid pain medications between 2000 and 2010 [5]. In this time period, the use of strong opioids increased in Switzerland by 100% [5]. The current study expands on these findings showing a further increase in prescriptions for all pain medications and in particular for metamizole, NSAIDs, Coxibs, paracetamol, and strong opioids. Similar to our study, an analysis of insurance claims data in Germany found an increased use of weak and strong opioids [24]. The increase was most pronounced for non-cancer pain conditions. A recently published population-based cohort study in Denmark linked legally prescribed opioids to an increased risk of deaths related to short- and long-term opioid use in patients with chronic non-cancer pain [25]. Wide geographical variations were also found in Germany for the use of metamizole [26]. One explanation may be that the assessment of an increased risk for agranulocytosis in metamizole users compared to other pain medications found in observational studies [27] varies. Missing safety data resulted in a ban in the USA and other countries [28] while metamizole remains a popular medication in other countries. A recent meta-analysis of the adverse effects of metamizole found that its short-term use in a hospital setting is safe [29]. However, insufficient evidence on the safety of long-term treatment is available [29].

Great effort was undertaken by the WHO to improve pain control in the world [4, 30]. The main objective was to identify and improve areas where pain management was insufficient. To date, current guidelines aim at early and aggressive pain treatment in different settings [31]. The use of strong opioids is well supported for the treatment of acute severe pain, for cancer pain, and for symptom relief at the end of life [32]. Here, the main concern is that patients living in certain areas are underserved and pain control is poor. In Switzerland, where compulsory health insurance secures access to health care, variations are more likely related to patient or physician preferences. The personal beliefs of patients and physicians may lead to reduced prescription of opioids in the treatment of cancer pain [33], for example. In chronic non-cancer pain, the efficacy of strong opioids is less well established and the risk for adverse events may outweigh the benefits [32, 34]. In chronic non-cancer pain it is unclear whether an increased use of strong opioids results in a better pain control. It is also unclear why in certain areas of Switzerland strong opioids are infrequently used and in others its use has recently increased by more than 200%.

In the past 20 years, a 14-fold increase in strong opioid use in the US was associated with an increased risk of unintentional opioid overdose [13–16]. In Europe, the increase in the use of opioids was less pronounced and the risk for opioid addiction is generally assumed to be low [35]. However, in a Danish cohort study the long-term use of strong opioids in chronic pain was associated with an increased risk for death and higher risks of injuries and toxicity/poisoning [25]. While in the US most overdose-related deaths were related to oxycodone, hydrocodone, and alprazolam [36], in Europe an increase of the illicit use of fentanyl was described and is best documented in Estonia [37]. Also in other European countries, the increased use of fentanyl has led to overdose-related deaths [37].

In most European countries, no surveillance system is in place that would detect an opioid overuse and unintentional overdose [38, 39]. Therefore, it is unclear whether an increase in unintentional opioid overdose or opioid misuse would be detected [40]. Similarly to other European countries, the increase in the use of strong opioids in the Swiss population was less pronounced than in the US. However, in certain areas, the use of strong opioids was well above the average. Explanatory factors and the potential risk for drug overdose are unclear.

### Strengths and limitations

To date, this is the first comprehensive analysis of insurance claims data examining the use of pain medication in the Swiss population. Great care was taken to assure the quality of the data extracted from the claims database. There are limitations that need further discussion. On the one hand analyses of health insurance databases on pain medications may overestimate the use of drugs as they are based on medications reimbursed. The effective quantity of medications used by patients may be lower, if pills are not actually taken. On the other hand, we have no information on medications sold over the counter (paracetamol, NSAIDs) and cross-border purchases. Therefore, the quantity of ingested non-opioid pain medications might be underestimated. We also were not able to discriminate between the use of drugs for pain or fever control and could not discriminate between the short- and long-term use of pain medications. We excluded opioids used within drug substitution programs. However, some medications including morphine, buprenorphine and methadone are used for both, pain treatment and opioid substitution. While we identified all records where the corresponding unique outpatient code was used, we cannot exclude that opioids were used for opioid dependency “off-label”. Whether the increase in the use of pain medication led to an increase in chronic use of pain medication is plausible but needs to be confirmed with future studies.

### Implication for research

The current analysis demonstrated an increase in the prescription of almost all pain medications. In particular, for strong opioids, physicians vary in their likelihood of prescribing by canton. Further research should explore the differences in the use of pain medications, in particular of strong opioids in cancer and non-cancer related diseases to reveal under- or overuse of particular medications. Further studies should investigate the incidence of the severe hematologic adverse effects caused by metamizole. Metimazole is a medication frequently used in the German speaking part of Switzerland.

## Conclusion

The analysis of insurance claims data provides evidence that the prescription rates for pain medications increased in Switzerland within the last ten years, in particular for metamizole and strong opioids. A further major finding was that the prescription rates for metamizole, paracetamol, and strong opioids varied substantially between different areas in Switzerland.

## Additional files

Additional file 1: ATC codes, route of administration and morphine equivalents for opioids. (DOC 72 kb)
Additional file 2: Change in claims for non-opioid pain medications, weak opioids, and strong opioids between 2006 and 2013. (DOC 132 kb)
Additional file 3: Reimbursement of claims for strong opioid substances in morphine equivalent dose (MED) and treatment days per 100’000 persons. (ZIP 187 kb)
Additional file 4: Geographical variation in increase of the use of strong opioids between 2006 and 2013 and variation in the use in 2006. **a** Increase between 2006 and 2013. Percent increase in the use of strong opioids per 100’000 persons stratified for age, sex, and cantons between 2006 and 2013. **b**: Geographical variation in the use of strong opioids in 2006. Strong opioids in 1000 mg MED per 100’000 persons in 2006 stratified for age, sex, and cantons. (ZIP 352 kb)

## Abbreviations

ATC codes: WHO pharmacological Anatomical Therapeutic Chemical (ATC) codes
Coxibs: Selective cyclooxygenase (COX)-2 inhibitors
DDD: Defined daily dose
MED: Morphine equivalent dose
mg: milligrams
NSAIDs: Non-steroidal anti-inflammatory drugs
WHO: World Health Organization.
