# Supplementary material for: Changes over time in prescription practices of pain medications in Switzerland between 2006 and 2013: an analysis of insurance claims
Source: BMC Health Serv Res. 2017 Feb 27;17:167. doi: 10.1186/s12913-017-2086-6 (PMC5327558; doi:10.1186/s12913-017-2086-6)
Supplement: Additional file 2: — Change in claims for non-opioid pain medications, weak opioids, and strong opioids between 2006 and 2013. (DOC 132 kb) [file 12913_2017_2086_MOESM2_ESM.doc]

Supplementary 2: Change in claims for non-opioid pain medications, weak opioids, and strong opioids between 2006 and 2013

| **Non-opioids** | ***2006*** | ***2007*** | ***2008*** | ***2009*** | ***2010*** | ***2011*** | ***2012*** | ***2013*** | ***% Diff 06-13*** |
| --- | --- | --- | --- | --- | --- | --- | --- | --- | --- |
|
| **Number of claims**: |  |  |  |  |  |  |  |  |  |
| Paracetamol: n | 512'762 | 559'845 | 609'804 | 646'843 | 567'118 | 593'953 | 616'901 | 639'716 | 25 |
| Metamizole: n | 55'000 | 69'521 | 90'137 | 103'931 | 118'181 | 141'895 | 167'296 | 185'168 | 237 |
| NSAID‡: n | 174'293 | 188'641 | 218'390 | 239'224 | 222'782 | 251'630 | 287'994 | 329'075 | 89 |
| Coxibs: n | 22'809 | 23'999 | 25'281 | 25'444 | 33'517 | 35'726 | 38'072 | 39'743 | 74 |
| **Claims / 100’000** |  |  |  |  |  |  |  |  |  |
| Paracetamol | 40'211 | 43'258 | 44'209 | 48'311 | 46'710 | 50'092 | 50'835 | 53'062 | 32 |
| Metamizol | 4'018 | 4'995 | 6'210 | 7'264 | 8'776 | 10'734 | 12'383 | 13'729 | 242 |
| NSAID + Coxibs | 15'634 | 16'913 | 18'170 | 20'402 | 22'207 | 25'574 | 28'196 | 31'994 | 105 |
| NSAIDs | 13'983 | 15'159 | 16'373 | 18'539 | 19'538 | 22'776 | 25'255 | 28'932 | 107 |
| Coxibs | 1'651 | 1'754 | 1'797 | 1'863 | 2'669 | 2'798 | 2'941 | 3'063 | 86 |
| Mefenamic acid | 13'758 | 13'434 | 13'054 | 12'992 | 12'494 | 12'218 | 11'378 | 10'922 | -21 |
| Diclofenac | 19'390 | 19'026 | 19'190 | 19'443 | 19'492 | 19'550 | 18'865 | 18'260 | -6 |
| Ibuprofen | 12'191 | 13'394 | 14'599 | 16'576 | 17'452 | 20'373 | 22'192 | 25'444 | 109 |
| Celecoxib | 1'651 | 1'754 | 1'797 | 1'739 | 1'688 | 1'663 | 1'567 | 1'509 | -9 |
| Etoricoxib |  |  |  | 123 | 981 | 1'135 | 1'374 | 1'554 |  |
| **Drug in 1000mg / 100'000** |  |  |  |  |  |  |  |  |  |
| Paracetamol | 1'023'125 | 1'183'648 | 1'330'349 | 1'536'066 | 1'667'246 | 1'820'224 | 1'915'959 | 2'041'595 | 100 |
| Metamizole | 53'694 | 69'574 | 89'281 | 109'084 | 127'126 | 162'354 | 198'925 | 228'921 | 326 |
| Ibuprofen | 235'285 | 246'540 | 262'146 | 278'319 | 299'714 | 332'623 | 356'881 | 395'328 | 68 |
| Mefenamic acid | 189'685 | 190'967 | 191'490 | 186'734 | 189'177 | 187'236 | 182'008 | 178'755 | -6 |
| Diclofenac | 39'882 | 39'294 | 40'124 | 40'152 | 40'927 | 40'659 | 39'616 | 39'075 | -2 |
| **Weak opioids** | ***2006*** | ***2007*** | ***2008*** | ***2009*** | ***2010*** | ***2011*** | ***2012*** | ***2013*** | ***% Diff 06-13*** |
|
| Number of persons† | 53'786 | 54'156 | 57'370 | 58'295 | 54'638 | 54'472 | 55'414 | 55'604 | 3 |
| Age: median (IQR) | 61 (29) | 61 (30) | 60 (29) | 60 (30) | 62 (29) | 62 (29) | 62 (29) | 62 (30) |  |
| Female: n (%) | 33'172 (62) | 33'337 (62) | 35'038 (61) | 35'585 (61) | 33'257 (61) | 33'161 (61) | 33'460 (60) | 33'629 (60) |  |
| Number of claims: n | 160'517 | 161'102 | 167'273 | 169'318 | 164'550 | 164'698 | 167'536 | 167'278 | 4 |
| Number of claims / person | 2.98 | 2.97 | 2.92 | 2.9 | 3.01 | 3.02 | 3.02 | 3.01 | 1 |
| Number of claims / 100'000 | 11'957 | 11'941 | 11'979 | 12'414 | 12'995 | 13'348 | 13'472 | 13'528 | 13 |
| Total MED in mg | 50'339 | 51'669 | 53'383 | 53'012 | 52'604 | 53'787 | 56'268 | 57'782 | 15 |
| Total MED in mg/Person | 936 | 954 | 931 | 909 | 963 | 987 | 1'015 | 1'039 | 11 |
| Total MED in 1000mg/ 100'000 | 3'768 | 3'843 | 3'841 | 3'911 | 4'159 | 4'322 | 4'476 | 4'655 | 24 |
| Tramadol in MED 1000mg/ 100'000 | 3'046 | 3'101 | 3'150 | 3'289 | 3'537 | 3'697 | 3'699 | 3'750 | 23 |
| Codeine MED mg/100'000 | 722 | 742 | 691 | 622 | 622 | 617 | 606 | 587 | -19 |
| Tapentadol MED 1000mg/100'000 | - | - | - | - | - | 9 | 170 | 317 | - |
| **Strong opioids** | ***2006*** | ***2007*** | ***2008*** | ***2009*** | ***2010*** | ***2011*** | ***2012*** | ***2013*** | ***% Diff 06-13*** |
|
| Number of persons | 16'744 | 19'207 | 20'851 | 22'190 | 23'165 | 25'081 | 27'278 | 28'509 | 70 |
| Age: median (IQR) | 66 (27) | 66 (27) | 67 (29) | 66 (29) | 68 (29) | 68 (29) | 68 (29) | 69 (29) |  |
| Female: n (%) | 10'297 (61.5) | 11'935 (62.1) | 12'957 (62.1) | 13'620 (61.4) | 14'058 (60.7) | 15'478 (61.7) | 16'775 (61.5) | 17'570 (61.6) |  |
| Number of claims#: n | 64'839 | 75'130 | 86'137 | 94'114 | 103'800 | 116'400 | 129'957 | 137'458 | 112 |
| Number of claims / person | 3.87 | 3.91 | 4.13 | 4.24 | 4.48 | 4.64 | 4.76 | 4.82 | 25 |
| Number of claims / 100'000 | 4'716 | 5'413 | 6'004 | 6'658 | 7'747 | 8'912 | 9'815 | 10'419 | 121 |
| Total MED in mg | 146'151 | 170'912 | 196'334 | 213'405 | 232'179 | 255'285 | 282'005 | 294'320 | 101 |
| Total MED in mg/Person | 8'729 | 8'898 | 9'416 | 9'617 | 10'023 | 10'178 | 10'338 | 10'324 | 18 |
| Total treatment days | 1'199 | 1'395 | 1'574 | 1'683 | 1'829 | 2'026 | 2'213 | 2'274 | 90 |
| Total treatment days/ person | 72 | 73 | 75 | 76 | 79 | 81 | 81 | 80 | 11 |
| Total MED in 1000mg/ 100'000 | 11'011 | 12'863 | 14'235 | 15'574 | 18'308 | 20'563 | 22'506 | 23'854 | 117 |
| Total treatment days / 100'000 | 89'903 | 104'947 | 114'177 | 122'716 | 140'855 | 160'758 | 173'468 | 180'371 | 101 |
| Total MED / treatment day | 121.9 | 122.5 | 124.8 | 126.8 | 126.9 | 126.0 | 127.4 | 129.4 | 6 |
| **Substances used*** |  |  |  |  |  |  |  |  |  |
| Morphine: | 2'875 | 3'333 | 3'257 | 3'218 | 3'429 | 3'864 | 4'370 | 4'648 | 62 |
| Oxycodone and comb: | 1'237 | 1'592 | 1'916 | 2'260 | 2'906 | 3'802 | 4'470 | 5'110 | 313 |
| Fentanyl: | 3'588 | 4'395 | 5'167 | 5'506 | 6'198 | 6'839 | 7'094 | 6'851 | 91 |
| Pethidine: | 33 | 30 | 38 | 29 | 32 | 45 | 53 | 39 | 18 |
| Buprenorphine | 2'699 | 2'853 | 3'137 | 3'562 | 4'435 | 4'564 | 5'240 | 6'049 | 124 |
| Hydromorphone | 548 | 651 | 710 | 924 | 1'040 | 915 | 756 | 730 | 33 |
| Methadone | 28 | 6 | 7 | 73 | 265 | 530 | 521 | 424 | 1414 |
| Nalbuphine: | 2 | 3 | 2 | 3 | 3 | 3 | 3 | 2 | 0 |
| **Treatment days^#^** |  |  |  |  |  |  |  |  |  |
| Morphine: | 28'988 | 33'642 | 32'849 | 32'434 | 34'528 | 38'905 | 44'046 | 46'856 | 62 |
| Oxycodone: | 10'994 | 14'154 | 17'034 | 20'091 | 25'831 | 33'794 | 39'735 | 45'421 | 313 |
| Fentanyl: | 29'941 | 36'644 | 43'125 | 45'968 | 52'036 | 57'364 | 59'664 | 57'953 | 94 |
| Pethidine: | 411 | 374 | 473 | 361 | 394 | 561 | 660 | 491 | 19 |
| Buprenorphine | 15'509 | 15'677 | 15'833 | 16'701 | 17'558 | 16'910 | 17'312 | 18'985 | 22 |
| Hydromorphone | 3'657 | 4'338 | 4'736 | 6'159 | 6'938 | 6'114 | 5'082 | 4'988 | 36 |
| Methadone | 373 | 86 | 97 | 969 | 3'533 | 7'070 | 6'937 | 5'645 | 1413 |
| Nalbuphine: | 31 | 31 | 30 | 31 | 36 | 39 | 35 | 30 | -3 |
| **Route of administration *** |  |  |  |  |  |  |  |  |  |
| Oral long acting: | 4'409 | 5'219 | 5'472 | 5'929 | 6'943 | 8'214 | 9'060 | 9'761 | 121 |
| Oral short acting: | 142 | 165 | 232 | 355 | 507 | 680 | 795 | 865 | 509 |
| Parenteral: | 168 | 229 | 228 | 219 | 228 | 272 | 319 | 328 | 95 |
| Rectal: | 5 | 4 | 3 | 2 | 2 | 2 | 6 | 9 | 80 |
| Sublingual: | 1'243 | 1'406 | 1'724 | 2'143 | 3'123 | 3'359 | 4'180 | 4'986 | 301 |
| Transdermal: | 5'045 | 5'841 | 6'575 | 6'925 | 7'506 | 8'036 | 8'146 | 7'905 | 57 |

† number of subjects that received reimbursement for one of the corresponding pain medications for a specific year

* substance used in 1000 mg (for opioids in MED/1000mg) extrapolated per 100’000 persons for the Swiss population based on the population insured by Helsana

# treatment days extrapolated per 100’000 persons of the Swiss population

‡ claims for all NSAID

MED, morphine equivalent dose
