# Supplementary figures and images for: Changes over time in prescription practices of pain medications in Switzerland between 2006 and 2013: an analysis of insurance claims
Source: BMC Health Serv Res. 2017 Feb 27;17:167. doi: 10.1186/s12913-017-2086-6 (PMC5327558; doi:10.1186/s12913-017-2086-6)

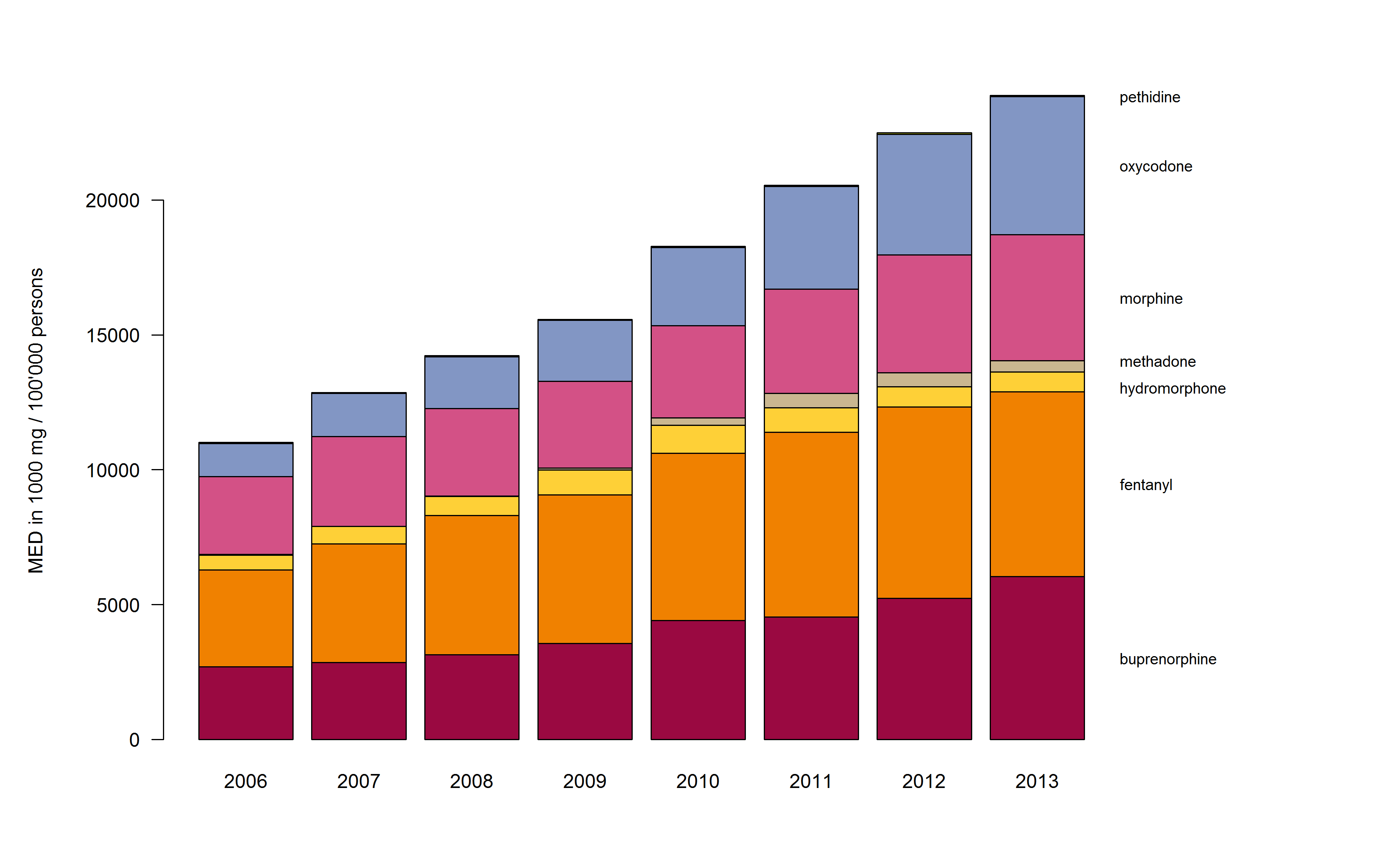

Supplement: Additional file 3: — Reimbursement of claims for strong opioid substances in morphine equivalent dose (MED) and treatment days per 100’000 persons. (ZIP 187 kb) [file 12913_2017_2086_MOESM3_ESM.zip › Appendix-3a-Strongs MED-2R3.tif]

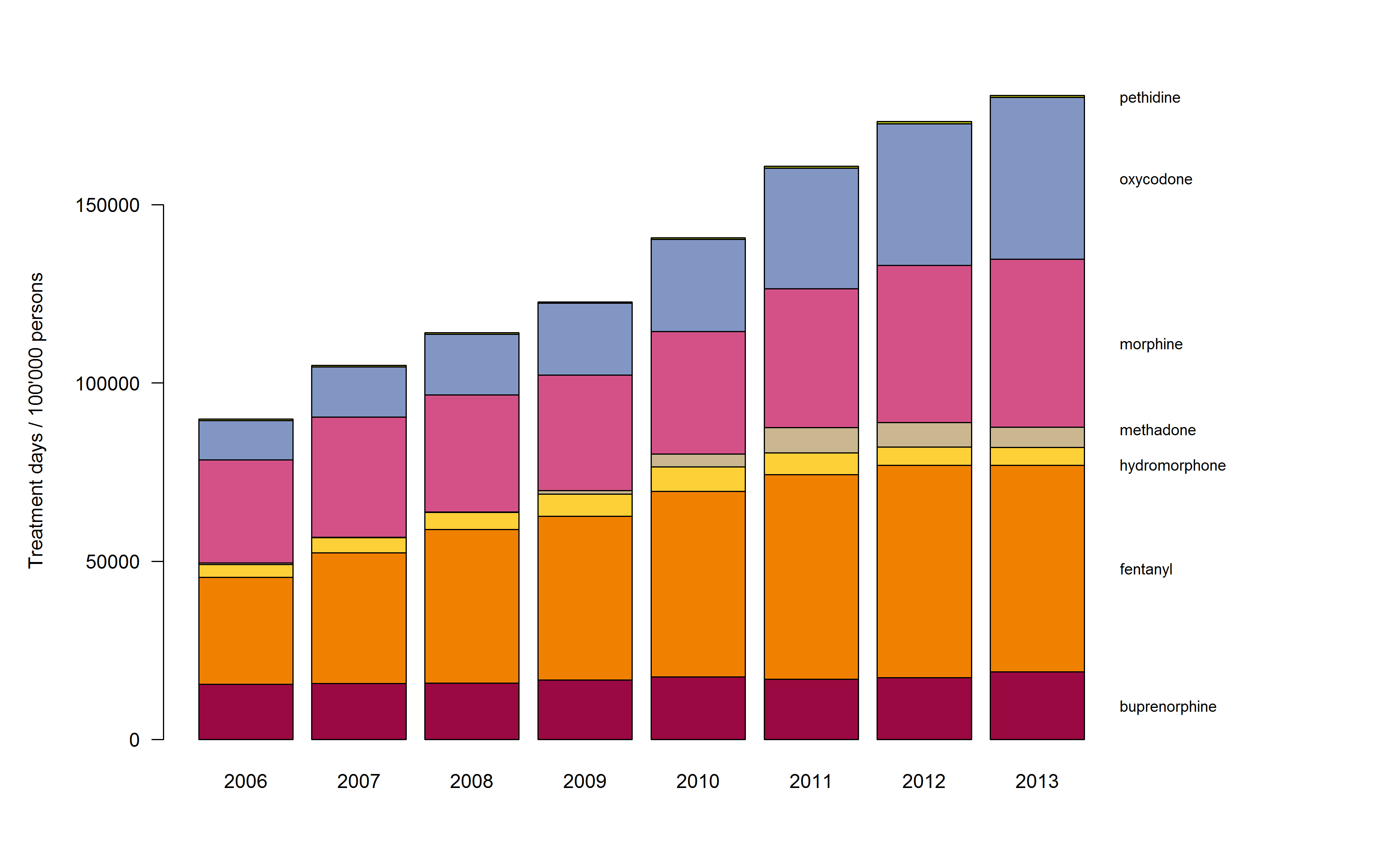

Supplement: Additional file 3: — Reimbursement of claims for strong opioid substances in morphine equivalent dose (MED) and treatment days per 100’000 persons. (ZIP 187 kb) [file 12913_2017_2086_MOESM3_ESM.zip › Appendix-3b-strongs Treatment days-2R3.tif]

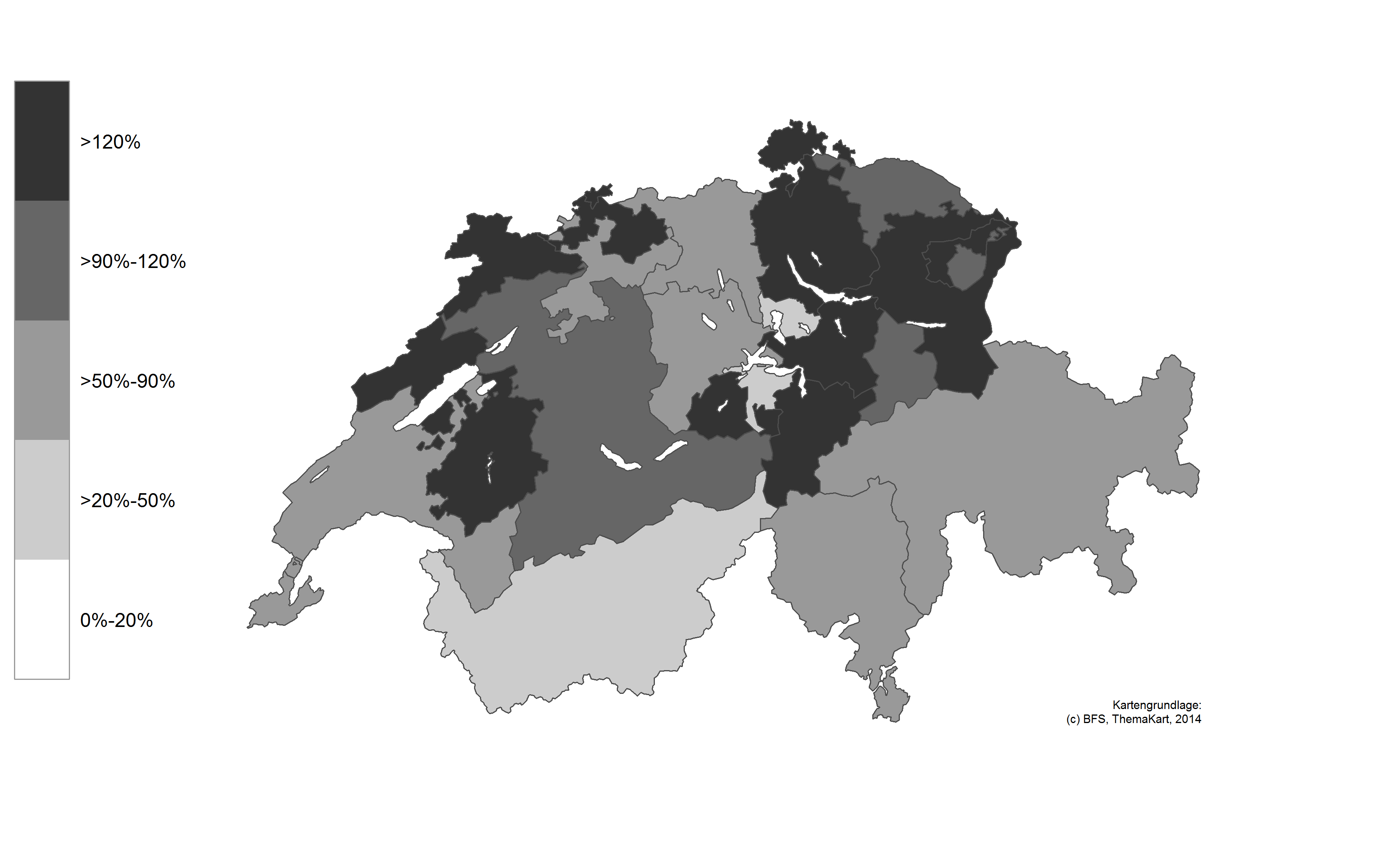

Supplement: Additional file 4: — Geographical variation in increase of the use of strong opioids between 2006 and 2013 and variation in the use in 2006. a Increase between 2006 and 2013. Percent increase in the use of strong opioids per 100’000 persons stratified for age, sex, and cantons between 2006 and 2013. b: Geographical variation in the use of strong opioids in 2006. Strong opioids in 1000 mg MED per 100’000 persons in 2006 stratified for age, sex, and cantons. (ZIP 352 kb) [file 12913_2017_2086_MOESM4_ESM.zip › Appendix-4a-Increase between 2006 and 2013-2R3.tif]

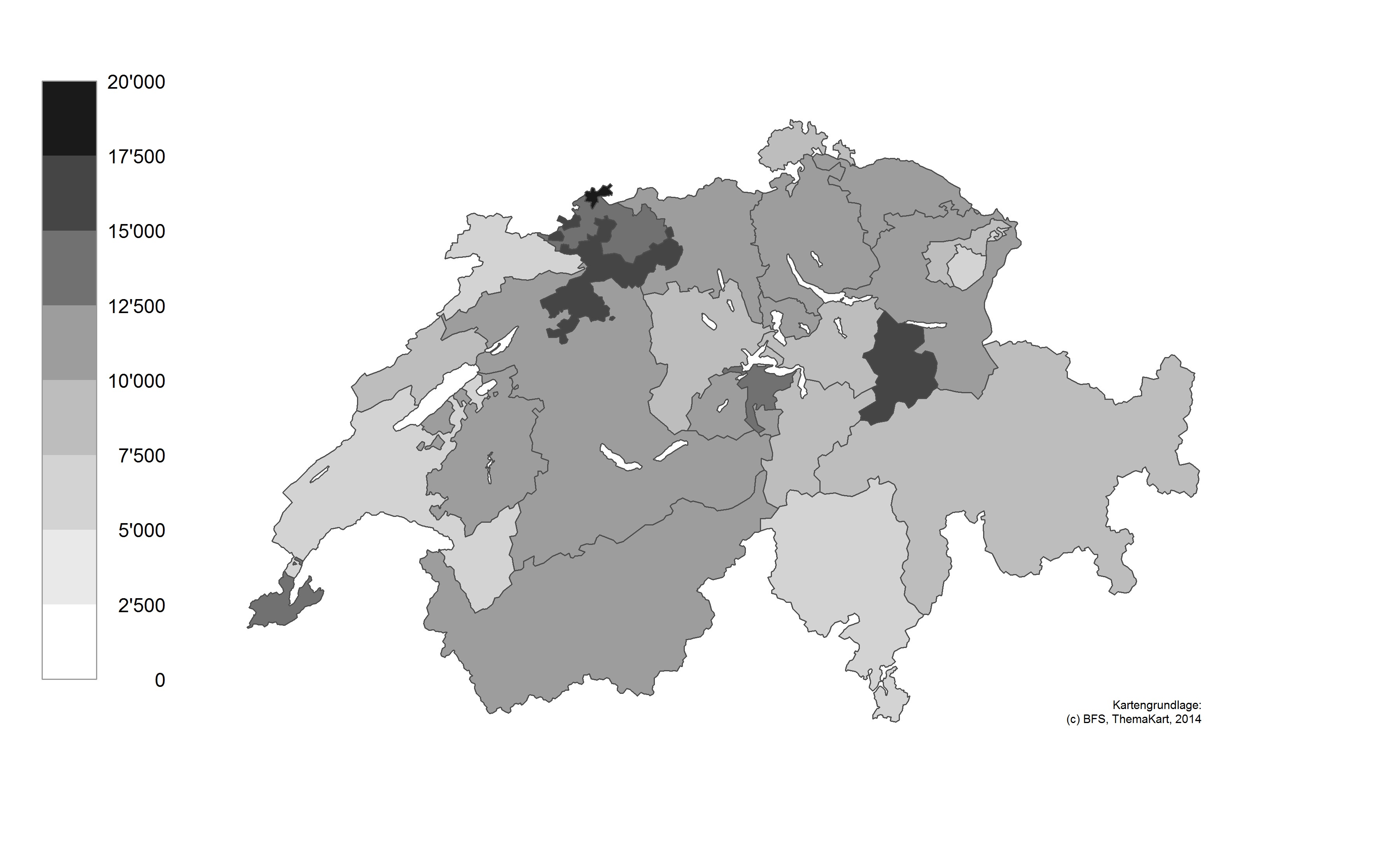

Supplement: Additional file 4: — Geographical variation in increase of the use of strong opioids between 2006 and 2013 and variation in the use in 2006. a Increase between 2006 and 2013. Percent increase in the use of strong opioids per 100’000 persons stratified for age, sex, and cantons between 2006 and 2013. b: Geographical variation in the use of strong opioids in 2006. Strong opioids in 1000 mg MED per 100’000 persons in 2006 stratified for age, sex, and cantons. (ZIP 352 kb) [file 12913_2017_2086_MOESM4_ESM.zip › Appendix-4b-strong-opioids-2006-2R3.tif]
